# Protocol summary of a randomized phase III study: comparing systemic therapy with and without debulking surgery (primary tumour resection) for clinical stage IVA (cT1-2bN0-1M1a) non-small cell lung cancer with radiologically undetermined pleural dissemination JCOG2103 (DEBULK-LUNG)

**DOI:** 10.1093/jjco/hyae143

**Published:** 2024-10-23

**Authors:** Yuta Sekino, Tomoyuki Hishida, Hiroshige Yoshioka, Masashi Wakabayashi, Noriko Mitome, Satoshi Shiono, Hirotsugu Kenmotsu, Kaname Nosaki, Keiju Aokage, Hidehito Horinouchi, Haruhiko Fukuda, Yuichiro Ohe, Shun-ichi Watanabe, the Lung Cancer Surgical Study Group (LCSSG) and the Lung Cancer Study Group (LCSG) of the Japan Clinical Oncology Group (JCOG)

**Affiliations:** Japan Clinical Oncology Group Data Center/Operations Office, National Cancer Center Hospital, Tokyo, Japan; Division of Thoracic Surgery, Department of Surgery, Keio University School of Medicine, Tokyo, Japan; Department of Thoracic Oncology, Kansai Medical University Hospital, Hirakata, Japan; Japan Clinical Oncology Group Data Center/Operations Office, National Cancer Center Hospital, Tokyo, Japan; Japan Clinical Oncology Group Data Center/Operations Office, National Cancer Center Hospital, Tokyo, Japan; Department of Surgery II, Faculty of Medicine, Yamagata University, Yamagata, Japan; Division of Thoracic Oncology, Shizuoka Cancer Center, Shizuoka, Japan; Department of Thoracic Oncology, National Cancer Center Hospital East, Chiba, Japan; Department of Thoracic Surgery, National Cancer Center Hospital East, Chiba, Japan; Department of Thoracic Oncology, National Cancer Center Hospital, Tokyo, Japan; Japan Clinical Oncology Group Data Center/Operations Office, National Cancer Center Hospital, Tokyo, Japan; Department of Thoracic Oncology, National Cancer Center Hospital, Tokyo, Japan; Department of Thoracic Surgery, National Cancer Center Hospital, Tokyo, Japan

**Keywords:** non-small cell lung cancer, pleural dissemination, debulking surgery

## Abstract

In patients with non-small cell lung cancer (NSCLC) who present with radiologically undetermined malignant pleural dissemination or incidental surgical diagnosis of the same, surgery is generally not the preferred option; systemic therapy is favoured. However, there is no consensus on incorporating primary site resection into the treatment plan. Retrospective analyses hint at potential benefits of combining systemic therapy with primary site resection, but prospective studies have yet to confirm these findings. Consequently, we have planned a multicentre, open-label, randomized controlled phase III trial to assess the efficacy of adding primary site resection to standard systemic therapy for stage IVA (cT1-2bN0-1M1a) NSCLC patients with radiologically undetermined pleural dissemination. The primary endpoint is overall survival. We aim to enroll 170 patients from 71 institutions over 5 years. This trial is registered at the Japan Registry of Clinical Trials (jRCT) under study number jRCTs031220666.

## Teaser

This multicentre, open-label, randomized controlled trial explores the efficacy of supplementing systemic therapy with surgery for NSCLC with radiologically undetermined pleural dissemination.

## Introduction

Non-small cell lung cancer (NSCLC) is a significant contributor to cancer-related mortality globally (1). Around 3% of lung cancer surgeries involve cases with pleural dissemination, undetected in pre-operative imaging and diagnosed during surgery (2). This radiologically undetermined pleural dissemination (RUPD) differs from apparent pleural dissemination, detectable in pre-operative images, in terms of tumour volume. Compared to apparent dissemination, RUPD has fewer metastatic tumours and necessitates pleural nodule biopsy for diagnosis confirmation. Despite this, the standard treatment for RUPD patients is systemic therapy, with surgery typically avoided due to the stage IVA (M1a) disease classification, regardless of metastatic volumes.

Advancements in systemic therapy, including molecular targeted agents and immune checkpoint inhibitors, have significantly improved the prognosis of stage IV NSCLC. However, original site failure accounts for 51.9% to 69.1% of progression, irrespective of pleural dissemination presence (3,4). As a result, there is growing demand for combining local therapy, including primary site surgery, with systemic therapy. This strategy aims for better local control and potential tumour burden reduction, preventing further pleural dissemination, malignant pleural effusion and distant metastasis, potentially extending survival. Retrospective studies have shown promising 3-year survival rates of 25% or higher (25.6%–44.4%), but these are limited by their retrospective nature, lack of modern regimen and potential selection bias regarding surgery timing and tumour factors (2,5–9).

Consequently, we designed a randomized controlled phase III trial to assess the efficacy of incorporating primary site resection into the standard systemic therapy for stage IVA (cT1-2bN0-1M1a) NSCLC with RUPD. RUPD patients are those slated for curative surgery but found to have pleural dissemination during surgery or those requiring surgical biopsy for definitive diagnosis due to equivocal small-sized pleural nodules on chest CT.

As for the timing of primary site resection, we opted for post-systemic therapy surgery rather than upfront surgery for three reasons: to prevent potential reduction in standard systemic therapy intensity or missed opportunities for standard systemic therapy due to surgery-related adverse events and performance status decline; to avoid enrolling patients with extremely poor prognosis due to initial drug resistance and to circumvent ineffective systemic therapy due to challenges in determining efficacy after upfront surgery; and to minimize patient burden, as upfront surgery would necessitate two surgeries in a short interval between the surgical confirmation of dissemination and the primary site resection after informed consent.

This study protocol, approved by the JCOG Protocol Review Committee in October 2022 and the Certified Review Board of National Cancer Center Hospital East in December 2022, was activated in February 2023. The trial, registered at the Japan Registry of Clinical Trials with the number jRCTs031220666 (https://jrct.niph.go.jp/latest-detail/ jRCTs031220666).

## Protocol digest of JCOG2103

### Purpose

We are conducting a phase III trial to ascertain the benefits of augmenting standard systemic therapy with primary tumour resection for clinical stage IVA (cT1-2bN0-1M1a) non-small cell lung cancer with radiologically undetermined pleural dissemination.

### Study setting

**Figure 1:**
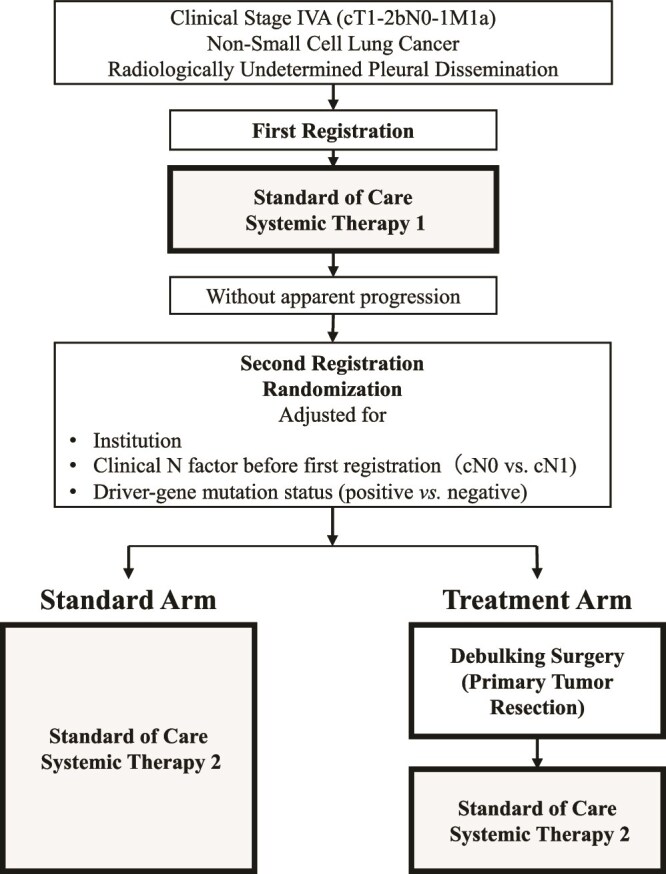
Study scheme.

This open-label, randomized controlled phase III trial involves 71 specialized centres. The study scheme is depicted in Fig. 1.

### Endpoints

The primary endpoint is overall survival (OS), defined as the time from randomization to death from any cause, censored on the last contact day for surviving patients. Secondary endpoints include progression-free survival, local progression-free survival, distant progression-free survival, cumulative incidence proportion of pleural effusion (Grade 2 or above), adverse events and quality of life (EQ-5D, FACT-L). Progression-free survival is defined as the duration from randomization to the first occurrence of disease progression, relapse or death from any cause and is censored at the last date of contact for a patient surviving without progression. Local progression free survival is the interval from randomization to the first instance of local progression or death from any cause, censored at the last contact date for a patient surviving without local progression. Distant progression free survival is the period from randomization to the first event of distant progression or death from any cause, censored at the last contact date for a patient surviving without distant progression. Adverse events are assessed according to the Common Terminology Criteria for Adverse Events version 5.0.

#### Inclusion criteria

Patients who meet all of the following criteria are enrolled in this trial:

### First registration criteria

(1) Pathological diagnosis of NSCLC by one of the following methods:
  i) Histological or cytological diagnosis of the primary tumour.
  ii) Histological diagnosis of pleural dissemination.
(2) Histological diagnosis of pleural dissemination lesion by thoracoscopic or open chest biopsy (positive pleural fluid cytology alone is not acceptable).
(3) The patient has cT1a-2bN0-1M1a clinical stage IVA (UICC TNM classification, 8th edition) with all of the following criteria (i) to (viii) on chest contrast-enhanced CT, thin slice CT, brain contrast-enhanced CT or magnetic resonance imaging, or integrated PET/CT:
  (i) No detectable pleural dissemination lesion on thin slice CT, or a present but unmeasurable pleural dissemination lesion (maximum diameter less than 10 mm).
  (ii) PET/CT shows no apparent abnormal FDG accumulation suspected as pleural dissemination in the pleura.
  (iii) No additional tumour nodule in the contralateral lung.
  (iv) No Grade 2 or higher pleural effusion.
  (v) No nodule is suspected as pericardial dissemination in the pericardium.
  (vi) No evidence of pericardial effusion exceeding physiological accumulation.
  (vii) No cN1 lymph node lesion with extranodal infiltration.
  (viii) Primary tumour and N1 lymph node (if cN1 is present) are deemed completely resectable through partial resection, segmental resection or lobectomy.
(4) *EGFR* mutations and *ALK* translocations are tested for non-squamous cell carcinoma.
(5) Age between 18 and 79 years at registration.
(6) Performance Status (ECOG) of 0 or 1.
(7) No prior thoracic surgery.
(8) No history of systemic therapy for lung cancer.
(9) No history of radiation therapy within the mediastinum or hilum for other cancers. Additionally, no history of Grade 2 or higher radiation pneumonitis.
(10) No active autoimmune disease or a history of chronic or recurrent autoimmune disease.
(11) No interstitial pneumonitis, pulmonary fibrosis or severe emphysema on chest CT.
(12) Predicted FEV1 after resection exceeds 800 ml.
(13) Sufficient organ function as follows:
  (i) White blood cell count ≥3000/mm^3^ and ≤ 12 000/mm^3^.
  (ii) Neutrophil count ≥1500/mm^3^.
  (iii) Haemoglobin level ≥ 9.0 g/dl.
  (iv) Platelet count ≥100 000/mm^3^.
  (v) Total bilirubin ≤2.0 mg/dl.
  (vi) Aspartate aminotransferase ≤100 IU/l.
  (vii) Alanine aminotransferase ≤100 IU/l.
  (viii) Serum creatinine ≤1.5 mg/dl.
  (ix) Creatinine clearance ≥45 ml/min/body.
  (x) SpO_2_ of ≥93% (room air).
(14) Written informed consent.

#### Exclusion criteria

Exclusion criteria: Patients meeting any of the following criteria are excluded from this trial:

1) Synchronous or metachronous (within five years) malignancies, excluding carcinoma in situ or intramucosal tumours treated curatively with local therapy, or early-stage cancers with a 5-year relative survival rate of ≥95%.
2) Infectious disease necessitating systemic treatment.
3) Body temperature exceeding 38° Celsius.
4) Females: pregnancy, within 28 days postparturition or lactation. Males: partners planning conception in the near future.
5) Presence of a psychological disorder that impedes participation in this clinical study.
6) Continuous systemic corticosteroid treatment with a prednisolone equivalent exceeding 10 mg/day, immunosuppressant treatment or immunoglobulin.
7) Unmanaged diabetes mellitus.
8) Uncontrolled arterial hypertension.
9) History of unstable angina pectoris within 3 weeks or myocardial infarction within 6 months prior to registration.
10) Uncontrolled valvular disease, dilated cardiomyopathy or hypertrophic cardiomyopathy.
11) Positive for HBs antigen or HIV antigen.

### Second registration criteria

1) Completion of systemic therapy:
  - (Driver gene mutation positive) Three courses of standard of care (SoC) are completed.
  - (Driver gene mutation negative) Four courses of SoC are completed, or only three courses are completed due to adverse events.
2) Fulfillment of response-related criteria:
  (i) Contrast-enhanced CT after the last course of SoC does not show progression compared to CT at first registration.
  (ii) Neither clinical nor radiological progression from the start of SoC.
3) Time from the last course of SoC:
  - (Driver gene mutation positive) Within 56 days from the start of the final course of SoC.
  - (Driver gene mutation positive) Within 48 days from the start of the final course of SoC.
4) Second registration occurs within 14 days of the response evaluation.
5) Time from the first registration is within 57–182 days.
6) The primary tumour is deemed completely resectable.
7) Predicted FEV1 after resection exceeds 800 ml.
8) Sufficient organ function is demonstrated as follows:
  (i) White blood cell count ≥2000/mm^3^
  (ii) Haemoglobin level ≥ 8.0 g/dl
  (iii) Platelet count ≥80 000/mm^3^
  (iv) Total bilirubin ≤2.0 mg/dl
  (v) AST ≤ 100 IU/l
  (vi) ALT ≤100 IU/l
  (vii) Serum creatinine ≤ 1.5 mg/dl
  (viii) SpO_2_ of ≥93% (room air).

### Randomization

Upon confirmation of the primary eligibility criteria, patients are registered via an electronic data capture to the JCOG Data Center. Following the verification of the second eligibility criteria, patients are randomized (1,1) to either the standard arm (standard of care systemic therapy [SoC]) or the treatment arm (debulking surgery with primary tumour resection and SoC). This is done using a minimization method with a random component, balancing the arms with the institution, the clinical N factor before first registration (cN0 vs. cN1) and the driver mutation status (positive vs. negative).

## Treatment methods

### Standard of care systemic therapy

The study’s protocol treatment regimen is outlined in Table 1. All patients receive the SoC after the first registration. For the driver mutation-positive group, patients with *EGFR* mutations receive a daily dose of 80 mg of osimertinib. Patients with *ALK* translocations are administered alectinib 300 mg twice daily. Those with *ROS1* mutations receive crizotinib 250 mg twice daily. Patients with *BRAF* (V600E) mutations undergo combination therapy of dabrafenib (150 mg twice daily) and trametinib (2 mg daily). For patients with *MET* exon 14 skipping mutations, a daily dose of 500 mg of tepotinib is administered. Lastly, patients with *RET* mutations receive selpercatinib 160 mg twice daily. One cycle is set for 4 weeks, as SoC1, with a total of three courses of SoC1 administered. After the second registration, the same targeted therapies continue unless there is unequivocal progression or unacceptable toxicities.

**Table 1:** Regimens of standard systemic therapy for care.

	Standard of care systemic therapy 1	Standard of care systemic therapy 2
**Driver mutation-positive**		
*EGFR* (ex19 del、L858R(±T790M))^a^	Osimertinib	
*ALK*	Alectinib	
*ROS1*	Crizotinib	
*BRAF* (V600E)	Dabrafenib + trametinib	
*MET* (ex14 skip)	Tepotinib	
*RET*	Selpercatinib	
**Driver mutation-negative**		
Non-squamous cell carcinoma	Carboplatin + pemetrexed + pembrolizumab	Pemetrexed + pembrolizumab
	Carboplatin + nab-paclitaxel + atezolizumab	Atezolizumab
Squamous cell carcinoma	Carboplatin + nab-paclitaxel + pembrolizumab OR carboplatin + paclitaxel + pembrolizumab	Pembrolizumab

^a^Note: If any unlisted *EGFR* gene mutations test positive, the case will be classified as *EGFR*-mutation negative.

For the driver mutation-negative group, patients with nonsquamous cell carcinoma receive either (i) carboplatin (CBDCA) AUC 5 (day 1) + pemetrexed 500 mg/m^2^ (day 1) + pembrolizumab 200 mg/body (day 1) every 3 weeks or (2) CBDCA AUC 6 (day 1) + nab-paclitaxel 100 mg/m^2^ (days 1, 8 and 15) + atezolizumab 1200 mg/body (day 1) every 3 weeks. Patients with squamous cell carcinoma receive either (1) CBDCA AUC 6 (day 1) + nab-paclitaxel 100 mg/m^2^ (days 1, 8 and 15) + pembrolizumab 200 mg/body (day 1) every 3 weeks or (2) CBDCA AUC 6 (day 1) + paclitaxel 200 mg/m^2^ (day 1) + pembrolizumab 200 mg/body (day 1) every 3 weeks. Each regimen is administered as SoC1, up to four cycles.

The duration of SoC1 was decided at 12 weeks (equivalent to three courses of 4-week cycles or four courses of 3-week cycles) for both driver mutation-positive and negative groups. In the case of driver mutation-positive cases, molecular targeted therapies typically show tumour shrinkage reaching a plateau at around 12 weeks (10). For driver mutation-negative cases, the standard combination immunotherapy is typically administered in 3-week cycles for a total of four cycles (12 weeks), after which treatment transitions to maintenance therapy with immune checkpoint inhibitor monotherapy. Anti-tumour effects can be sufficiently confirmed within these four courses (12 weeks). Moreover, meta-analysis has shown that administering more than four courses does not significantly extend survival (11).

After the second registration, the same immune checkpoint inhibitors are continued as maintenance therapy, unless there is unequivocal progression or unacceptable toxicities. Patients with nonsquamous cell carcinoma receive either (1) pemetrexed 500 mg/m^2^ (day 1) + pembrolizumab 200 mg/body (day 1) every 3 weeks or (2) atezolizumab 1200 mg/body (day 1) every 3 weeks. Patients with squamous cell carcinoma receive pembrolizumab 200 mg/body (day 1) every 3 weeks. SoC2 treatment initiation is scheduled using the surgical date as the reference point (day 0), with the driver mutation-positive group to start SoC2 within 7–28 days post-operation and the negative group within 7–56 days.

### Debulking surgery

The surgical procedure can be executed via an open approach, thoracoscopic approach (including assisted techniques) or robot-assisted approach. The primary tumour is removed through either a wedge resection or segmentectomy. However, lobectomy is allowed when sublobar resection is deemed unfeasible due to the presence of cN1 or insufficient margin. If the patient shows no clinical evidence of lymph node involvement at the time of initial registration, neither lymph node sampling nor dissection is performed. If the patient has cN1 at the time of initial registration, the relevant lymph nodes are dissected. The decision to resect remaining disseminated lesions and the extent of resection are determined based on the surgeon’s discretion and the size of the remaining disseminated lesions.

### Follow-up

All enrolled patients undergo a 5-year follow-up. Chest and abdominal CT scans are regularly evaluated. During the first 2 years post protocol treatment initiation, patients with a positive driver mutation are evaluated every 8 weeks, while those with a negative mutation are evaluated every 9 weeks. After 2 years, evaluations are conducted every 12 weeks, and after 5 years, every 24 weeks, regardless of their driver mutation status. Patient-reported outcomes are assessed using the EQ-5D (EuroQol 5-dimension) and FACT-L (Functional Assessment of Cancer Therapy-Lung). These assessments are conducted at the second registration and at 6, 12 and 24 months post second registration.

### Study design and statistical analysis

This multi-institutional, randomized controlled trial is designed to validate the superiority of the treatment arm (which adds debulking surgery to SoC) over the control arm (SoC alone), with the primary endpoint being OS, in all patients enrolled for second registration. We assumed a 3-year OS of 50% in the control arm and expect a 15% improvement in the treatment arm. The required sample size, calculated using Schoenfeld and Richter’s method (12), is a total of 140 patients (70 patients per arm), considering a one-sided alpha of 5%, a power of 70%, an accrual period of 5 years, and a follow-up period of 3 years. The total sample size is set at 170 to account for potential loss to follow-up and progression during SoC1 before second registration.

OS will be compared using the stratified log-rank test, with clinical N factors and driver mutation status as strata. The stratified Cox proportional hazards model will be employed to estimate the hazard ratio between the arms. All statistical analyses will be conducted at the JCOG Data Center.

### Interim analysis and monitoring

Two interim analyses are scheduled for this study. The first will take place after half of the planned number of patients have been enrolled, and the second will occur once the planned patient accrual is achieved, and the patients’ protocol treatment is completed. The Lan–DeMets α-spending function, in conjunction with the O’Brien and Fleming types, will be utilized to adjust for multiplicity (13). If the primary endpoint is higher in the perioperative chemotherapy arm than in the post-operative chemotherapy arm, with a *p*-value less than the adjusted significance level, the study may be considered for termination due to efficacy. The conditional power (14) and predictive probability (15) for the primary endpoint will be employed to determine whether the study should be terminated due to futility. The JCOG’s Data and Safety Monitoring Committee will independently review the interim analysis report, separate from the group investigators and group statistician.

## Figures and Tables

**North to South:** 

No	LCSSG	LCSG	
1		X	Asahikawa Medical Center
2		X	National Hospital Organization Hokkaido Cancer Center
3		X	Hokkaido University Hospital
4	X		National Hospital Organization, Sendai Medical Center
5	X		Tohoku University Hospital
6		X	Miyagi Cancer Center
7		X	Sendai Kousei Hospital
8		X	Akita Kousei Medical Center
9	X		Yamagata Prefectural Central Hospital
10	X		Yamagata University Hospital
11	X		Ibaraki Prefectural Central Hospital & Cancer Center
12	X	X	Tochigi Cancer Center
13		X	Dokkyo Medical University Hospital
14	X	X	Gunma Prefectural Cancer Center
15		X	Saitama Cancer Center
16	X	X	National Cancer Center Hospital East
17	X		Chiba University, Graduate School of Medicine
18		X	Funabashi Municipal Medical Center
19	X	X	National Cancer Center Hospital
20	X		Kyorin University Faculty of Medicine
21	X		Tokyo Medical University Hospital
22	X	X	Tokyo Metropolitan Cancer and Infectious diseases Center Komagome Hospital
23		X	National Center for Global Health and Medicine (NCGM)
24	X		Keio University Hospital
25	X	X	Showa University School of Medicine
26	X	X	Jikei University Hospital
27	X	X	Cancer Institute Hospital of Japanese Foundation for Cancer Research
28	X		The University of Tokyo Hospital
29	X	X	Juntendo University Hospital
30		X	Toho University Omori Medical Center
31	X	X	Nippon Medical School Hospital
32	X		St.Marianna University School of Medicine
33	X	X	Kanagawa Cancer Center
34	X	X	Yokohama Municipal Citizen’s Hospital
35	X	X	Kitasato University School of Medicine
36	X	X	Niigata Cancer Center Hospital
37		X	Niigata University Medical and Dental Hospital
38	X		Kanazawa University School of Medicine
39		X	Shinshu University School of Medicine
40	X		Gifu University School of Medicine
41	X	X	Shizuoka Cancer Center
42	X	X	Aichi Cancer Center Hospital
43	X	X	Nagoya University School of Medicine
44		X	Fujita Health University
45	X	X	Kyoto University Hospital
46	X		Osaka University Graduate School of Medicine
47	X	X	Osaka Metropolitan University Hospital
48	X	X	Kindai University Hospital
49	X	X	Osaka International Cancer Institute
50	X	X	Osaka City General Hospital
51	X	X	Kansai Medical University Hospital
52	X		Kobe University Graduate School of Medicine
53		X	Hyogo Medical University
54		X	Itami City Hospital
55		X	Wakayama Medical University, School of Medicine
56	X	X	Kurashiki Central Hospital
57	X		Okayama University Hospital
58		X	Japanese Red Cross Okayama Hospital
59	X		National Hospital Organization Kure Medical Center Chugoku Cancer Center
60	X	X	Hiroshima University Hospital
61	X		Hiroshima City Hiroshima Citizens Hospital
62		X	National Hospital Organization Yamaguchi-Ube Medical Center
63	X	X	National Hospital Organization Shikoku Cancer Center
64	X	X	National Kyushu Cancer Center
65		X	National Hospital Organization Kyushu Medical Center
66		X	Kurume University School of Medicine
67	X		University of Occupational and Environmental Health
68	X	X	Kyushu University Hospital
69	X	X	Nagasaki University Hospital
70	X	X	Kumamoto University Hospital
71	X		Oita University Hospital

## jRCT registration number

This trial has been registered at the Japan Registry of Clinical Trials as jRCTs031220666 (https://jrct.niph.go.jp/latest-detail/jRCTs031220666).
